# Transport of Corilagin, Gallic Acid, and Ellagic Acid from Fructus Phyllanthi Tannin Fraction in Caco-2 Cell Monolayers

**DOI:** 10.1155/2016/9205379

**Published:** 2016-09-21

**Authors:** Xin Mao, Ling-Fang Wu, Hai-juan Zhao, Wen-Yi Liang, Wen-Jing Chen, Shu-Xian Han, Qi Qi, Ya-Ping Cui, Shi Li, Guang-Hui Yang, Yan-Yan Shao, Dan Zhu, Ru-Feng Wang, Yun You, Lan-Zhen Zhang

**Affiliations:** ^1^School of Chinese Materia Medica, Beijing University of Chinese Medicine, Beijing 100102, China; ^2^Institute of Chinese Materia Medica, China Academy of Chinese Medical Sciences, Beijing 100700, China; ^3^Key Laboratory of Chinese Internal Medicine, Beijing University of Chinese Medicine, Beijing 100700, China

## Abstract

*Objective*. To investigate the absorption property of the representative hydrolyzable tannin, namely corilagin, and its hydrolysates gallic acid (GA) and ellagic acid (EA) from the Fructus Phyllanthi tannin fraction (PTF)* in vitro*.* Methods*. Caco-2 cells monolayer model was established. Influences of PTF on Caco-2 cells viability were detected with MTT assay. The transport across monolayers was examined for different time points, concentrations, and secretory directions. The inhibitors of P-glycoprotein (P-gp), multidrug resistance proteins (MRPs), organic anion transporting polypeptide (OATP) and sodium/glucose cotransporter 1 (SGLT1), and tight junction modulators were used to study the transport mechanism. LC-MS method was employed to quantify the absorption concentration.* Results*. The apparent permeability coefficient (*P*_app_) values of the three compounds were below 1.0 × 10^−6^ cm/s. The absorption of corilagin and GA were much lower than their efflux, and the uptake of both compounds was increased in the presence of inhibitors of P-gp and MRPs. The absorption of EA was decreased in the company of OATP and SGLT1 inhibitors. Moreover, the transport of corilagin, GA, and EA was enhanced by tight junction modulators.* Conclusion*. These observations indicated that the three compounds in PTF were transported via passive diffusion combined with protein mediated transport. P-gp and MRPs might get involved in the transport of corilagin and GA. The absorption of EA could be attributed to OATP and SGLT1 protein.

## 1. Introduction

Hydrolyzable tannins belong to the family of polyphenol, which integrated in some kinds of foods and medicinal herbs, such as* Punica granatum* and *Terminalia chebula* Retz. Recently, researches of hydrolyzable tannins are getting more and more attentions on account of their benefits for our health [1]. As a typical hydrolyzable tannin, corilagin is a potential antitumor compound [2, 3]. In addition, it exhibits antiviral, antihyperalgesic, and antibacterial activities [4–6]. Gallic acid (GA) and ellagic acid (EA) are two hydrolysates of corilagin, both of which possess strong antioxidant and anticancer effects [7–10].

*Phyllanthus emblica L.* (Euphorbiaceae) is a famous medicinal plant distributed in Asia countries especially in China. Its fruit, Fructus Phyllanthi, has been used for the treatment of liver and digestive diseases in China for a long history [11, 12]. The recent studies of our team demonstrated that Fructus Phyllanthi tannin fraction (PTF) inhibits the migration and invasion of human lung squamous carcinoma cell line (NCI-H1703)* in vitro* through MAPK/MMP pathways [13]. Corilagin, EA, and GA are the three main compounds, which are contained about 10.26% in PTF [14] and the biological effects of PTF are considered to be correlated with these compounds. In order to elucidate the absorption of PTF, an* in vitro* Caco-2 monolayer model was established.

Caco-2 cells were derived from human colorectal carcinoma, and Caco-2 cell monolayer is the classical cell model of intestinal epithelium [15, 16]. There are various transporters expressed in the membranes such as P-gp, MRPs, OATP, SGLT1, monocarboxylic acid transporters (MCT), and breast cancer resistance protein (BCRP) [17–20]. Intestinal absorption of polyphenol compounds, most of which were flavonoids, across Caco-2 cell monolayer was reported by various researchers [21–23]. These flavonoids such as apigenin, orientin, and (-)epicatechin-3-gallate were hardly transported through the monolayer and possibly via paracellular pathway, and some of them were suggested to be mediated by carrier protein [21–23].

Our previous studies found that the absorption of corilagin, EA, and GA was very poor with peak concentration values (*C*_max_) lower than 10.47 *μ*g/mL after oral administration of PTF at dose of 6 g/kg in rats [14]. It is essential to find out why the low absorption of oral PTF occurred and to identify suitable pharmaceutical methods to improve the absorption of PTF and further enhance the anticancer activities. P-gp inhibitor (verapamil) [21, 22] and MRPs inhibitor (indomethacin) [24, 25] were employed to investigate whether the transport was affected by the efflux transporters, OATP inhibitor (indomethacin) [26, 27] and SGLT1 inhibitor (phloridzin) [21, 28] were chosen to study the effect of absorption transporters, and tight junction modulators (sodium dodecyl sulfate (SDS) and poly-l-lysine (PLL)) [29, 30] were used to evaluate the role of paracellular permeability in the transport of these three compounds from PTF.

## 2. Materials and Methods

### 2.1. Materials

Corilagin, GA, EA, and fluorescein sodium were purchased from National Institute for the Control of Pharmaceutical and Biological Products (Beijing, China). Phloridzin, indomethacin, verapamil, SDS, and type I collagen (from rat tail) were obtained from Sigma Chemical Co. (St. Louis, MO, USA). PLL (MW: 70,000–150,000) was purchased from ScienCell (Carlsbad, CA, USA). Dulbecco's modified Eagle's minimal essential medium (DMEM) and penicillin-streptomycin were from Thermo (Beijing, China). Fetal bovine serum (FBS), nonessential amino acid (NEAA), and Hank's balanced salt solution (HBSS) came from Gibco Life Technologies (Grand Island, NY, USA). 12-well Transwell-Clear plates (pore size 0.4 *μ*m, surface area 1.12 cm^2^) and 96-well plates were from Corning Incorporated.

### 2.2. Extraction and Purification of PTF

The fruit of* Phyllanthus emblica L.* (Fructus Phyllanthi) grown in Nepal was purchased from the Beijing Tibetan Hospital. The powder of Fructus Phyllanthi was extracted three times through reflux at 60°C with ethanol/water (3 : 2; v/v), and the extract was combined and concentrated with rotary evaporator (EYELA, NN Series; Rikakikai Co. Ltd., Tokyo, Japan) at 40°C. The supernatant of condensed liquid was collected by centrifugation and enriched by gradient elution on macroporous resin column. The content of total tannins of PTF determined by tungsten molybdophosphate-casein colorimetric method was higher than 56%. The HPLC fingerprint of the PTF was studied, from which three main peaks were identified as GA, corilagin, and EA [31]. They were quantitatively analyzed using RP-HPLC method, and their concentrations were 4.72% ± 0.06%, 1.87% ± 0.05%, and 3.67% ± 0.04%, respectively [14]. The PTF was dissolved and diluted with HBSS solution to a final concentration.

### 2.3. Cell Culture

Caco-2 cell line was derived from American Type Culture Collection (Manassas, VA, USA). Caco-2 cells were grown in DMEM, supplemented with 10% FBS, 1 mM NEAA, 100 U/mL penicillin, and 0.1 *μ*g/mL streptomycin and cultured in a humidified atmosphere of 5% CO_2_ in air at 37°C.

The type I collagen dissolved in 0.1 M acetic acid was used to coat Transwell-Clear plates at 8 *μ*g/cm^2^; the supernatant was removed after binding for 2 hours at 37°C. The plates were washed twice with phosphate buffered saline and dried overnight.

When cells were cultured to 80% confluence, they were seeded in 12-well Transwell-Clear plates at a density of 1.2 × 10^5^ cells/insert. Cells were cultured for 21 days to reach confluence and functional differentiation. Complete medium was replaced every 2 days for the first 2 weeks and daily renewed for the last 7 days. All cells were between passages 35 and 55. Transepithelial electrical resistance (TEER) values were tested using Millicell ERS-2 Volt-Ohm Meter (Millipore, Billerica, MA, USA), and the TEER values >500 Ω/cm^2^ were required.

### 2.4. Cell Viability Evaluation

MTT assay was employed to test the influence on cell viability of PTF on Caco-2 cells. Caco-2 cells were seeded in 96-well plate at a density of 2 × 10^4^ cells/well and cultured until confluence. Then, the DMEM culture medium was replaced with 150 *μ*L HBSS and incubated with different concentrations of fractions for 4 h. The concentration of PTF was ranged from 0.25 to 2 mg/mL. Thereafter, the HBSS was removed and the monolayers were incubated with 200 *μ*L of DMEM culture medium containing MTT 500 *μ*g/L for 4 h. Finally, the medium was removed, the formazan salt crystals that remained were dissolved with 150 *μ*L of DMSO and shaken for 10 min, and the absorbance was measured at 570 nm using microplate reader (Molecular Devices, Sunnyvale, CA, USA) [28]. The cell inhibitory rate was calculated as follows: inhibitory rate (%) = (A570_control_ − A570_sample_)/(A570_control_ − A570_blank_) × 100%.

### 2.5. Transport Experiments

After 21-day culture, the complete medium was removed and the monolayer was washed three times and balanced with HBSS (previously warmed to 37°C) for 30 min. PTF (0.25, 0.50, and 1.00 mg/mL) was added onto the Apical side (0.5 mL) or Basolateral side (1.5 mL), while blank HBSS was added onto the receiving chamber. Aliquots of 200 *μ*L samples were taken from the receiving chamber every 30 min for 180 min and each time an equal volume of blank HBSS was replenished. All samples were dried with organomation. Before analyzing, the dry samples were dissolved in methanol/water/acetic acid (200 : 200 : 1, v/v/v) and the insoluble substance was eliminated with centrifugation 12000 rpm for 5 min.

Protein inhibitors including verapamil (100 *μ*M), indomethacin (10 *μ*M), and phloridzin (50 *μ*M) were employed to investigate the role of different transporters. Epithelial tight junction modulators such as SDS (50 *μ*g/mL) and PLL (50 *μ*g/mL) were used to evaluate the role of paracellular permeability.

Hydrophilic marker sodium fluorescein (0.6 mg/mL) was used for the evaluation of the integrity of the cell monolayer. The absorbance of the marker was measured at 490 nm using the microplate reader (Molecular Devices, Sunnyvale, CA, USA), and the concentration of sodium fluorescein was calculated by standard curve. The *P*_app_ for the hydrophilic marker was required lower than 1.00 × 10^−6^ cm/s.

### 2.6. LC-MS Analysis

The analytes were analyzed on a Diamonsil C18 column (250 × 4.6 mm, 5 *μ*m i.d., Dikma Technologies Inc., CA, USA) using an Agilent 1260 series liquid chromatograph (Agilent Technologies, CA, USA) comprised of an auto injector, a quaternary pump, a diode array detector (DAD), and a column counterpart. The elution was performed with mobile phase composed of 0.1% formic acid in acetonitrile (solvent A) and 0.1% formic acid in water (solvent B) using the following gradient program at the flow rate of 1 mL/min: 0–12 min, linear gradient 15–65% (A); 12-13 min, linear gradient 65–100% (A); 13–25 min, isocratic gradient 100-100% (A). The column temperature was 20°C and the injection volume was 20 *μ*L.

MS/MS analysis was acquired using an Agilent 6410 triple quadrupole mass spectrometer (Agilent Technologies, CA, USA) equipped with an electrospray ionisation (ESI) in the negative mode. Other analytical conditions included the drying gas temperature of 300°C, the drying gas flow of 11 L/min, the nebulizer pressure of 35 psi, and the capillary voltage of 4 kV. To get the highest abundance, the fragmentor voltage and collision energy were optimized. Data acquisition and processing were accepted by Agilent Mass Hunter workstation software version B.05.00 using multiple reaction monitoring (MRM). The quantification was performed using external standard method.

### 2.7. Data Analysis

The *P*_app_ and PDR were determined according to the equation:

(1) Papp=dC/dt×VA×C0,PDR=PappBL→APPappAP→BL.

Here, d*C*/d*t* is the change in concentration on the receiver side over time; *V* is the volume of the solution in the receiver chamber; *A* is the surface area of the cell monolayer; *C*_0_ is the initial concentration in the donor chamber. PDR is the ratio of *P*_app(BL→AP)_ to *P*_app(AP→BL)_.

The results presented in this study were the averages of at least three replicates and were expressed as the mean ± SEM. Differences amongst treatments were detected by analysis of one-way ANOVA. The differences were considered to be significant when *P* < 0.05.

## 3. Results

### 3.1. Validation of the Caco-2 Monolayers

The TEER values of the monolayers increased over the first 12 days and steadily for the last 9 days. The values were above 500 Ω/cm^2^ on day 21. The *P*_app_ value of sodium fluorescein, which is a poorly transported marker, tested across the monolayers was 3.16 ± 0.25 × 10^−7^ cm/s which agreed with that published in the previous literature [32, 33].

### 3.2. Cell Viability Evaluation

**Figure 1:**
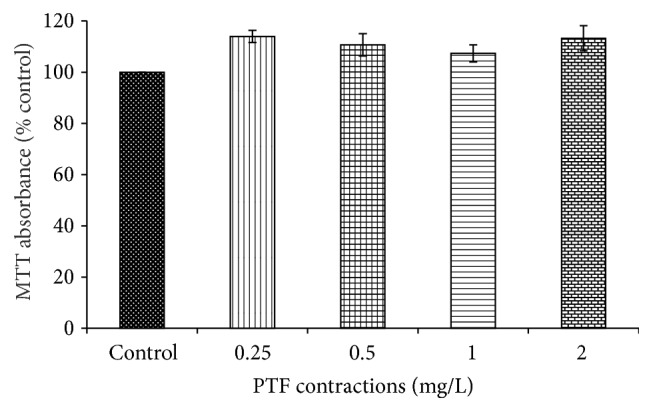
Effects of PTF on viability of Caco-2 cells. Results are expressed as the mean ± SEM for 10 determinations.

At the concentrations of 0.25, 0.50, 1.00, and 2.00 mg/mL, no significant effects on growth of Caco-2 cells were observed in 4 h (Figure 1), which indicated that PTF exhibited no toxic to Caco-2 cells at 0.25–2.00 mg/mL for 4 h.

### 3.3. Quantification of Analytes in Herbal Extracts by LC-MS

**Figure 2:**
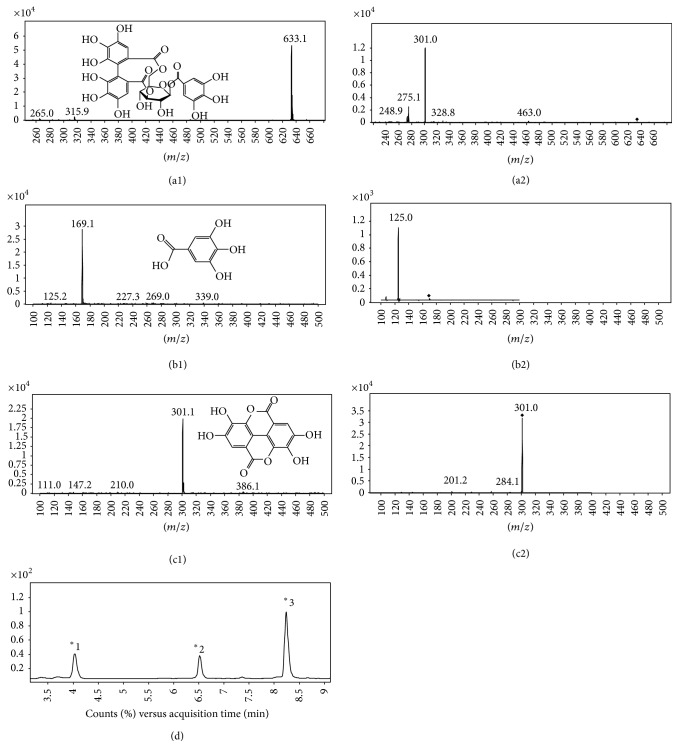
Structure and mass spectra of corilagin, GA, and EA and typical LC-MS chromatogram of PTF in HBSS solution. Scan spectra (a1, b1, and c1) and product ion (a2, b2, and c2) of corilagin, GA, and EA were got in negative ion mode. In the typical LC-MS chromatogram (d), peaks ^*∗*^1, ^*∗*^2, and ^*∗*^3 correspond to GA, corilagin, and EA in PTF with concentration of 5 *μ*g/mL and the chromatogram was using TIC mode. The diamond shape refers to the precursor ion (M-1) of the product ion.

The MS conditions of LC-MS method had been optimized to achieve the highest abundance of corilagin, GA, and EA when the MRM mode was used and the [M − 1]^−^ was employed as the precursor ion (Figure 2). For corilagin the retention time was 6.61 min, the mass transition used was* m/z* 633.1→301.0 (collision energy, −35 eV), the linear equation was *Y* = 23.705*X* − 26.106  (*r* ^2^ = 0.9999) with a good linearity over the range from 0.65 ng/mL to 653 ng/mL, the limit of detection (LOD, signal-to-noise ratio ≥ 3) was 1.35 ng/mL, and the limit of quantitation (LOQ, signal-to-noise ratio ≥ 10) was 1.94 ng/mL. For GA the retention time was 4.14 min, the mass transition used was* m/z* 169.0→125.0 (collision energy, −10 eV), the linear equation was *Y* = 28.769*X* + 22.697  (*r* ^2^ = 0.9999) with a good linearity over the range from 1.01 ng/mL to 252 ng/mL, the LOD was 0.08 ng/mL, and the LOQ was 1.64 ng/mL. For EA the retention time was 8.39 min, the mass transition used was* m/z* 301.0→301.0 (collision energy, −10 eV), the linear equation was *Y* = 111.21*X* + 130.99  (*r* ^2^ = 0.9982) with a good linearity over the range from 1.98 ng/mL to 989 ng/mL, the LOD was 0.17 ng/mL, and the LOQ was 0.97 ng/mL.

Three concentrations of corilagin, GA, and EA in six replicates in a single day were analyzed as intraday precision and duplicating the intraday experiment on two successive days was analyzed as interday precision. The precision and recovery data are shown in Table 1.

### 3.4. Transport of PTF across Caco-2 Cell Monolayers

**Figure 3:**
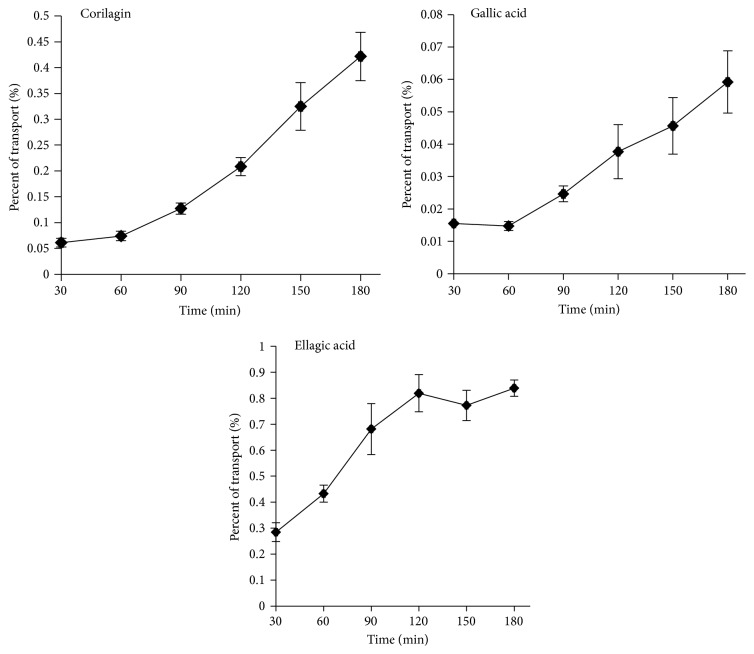
The percent transport of the uptake of three compounds in PTF across Caco-2 cell monolayers at different time points during 180 min. Caco-2 cells were incubated with PTF at the concentration of 1.00 mg/mL. Results are expressed as the mean ± SEM for 3 determinations.

**Figure 4:**
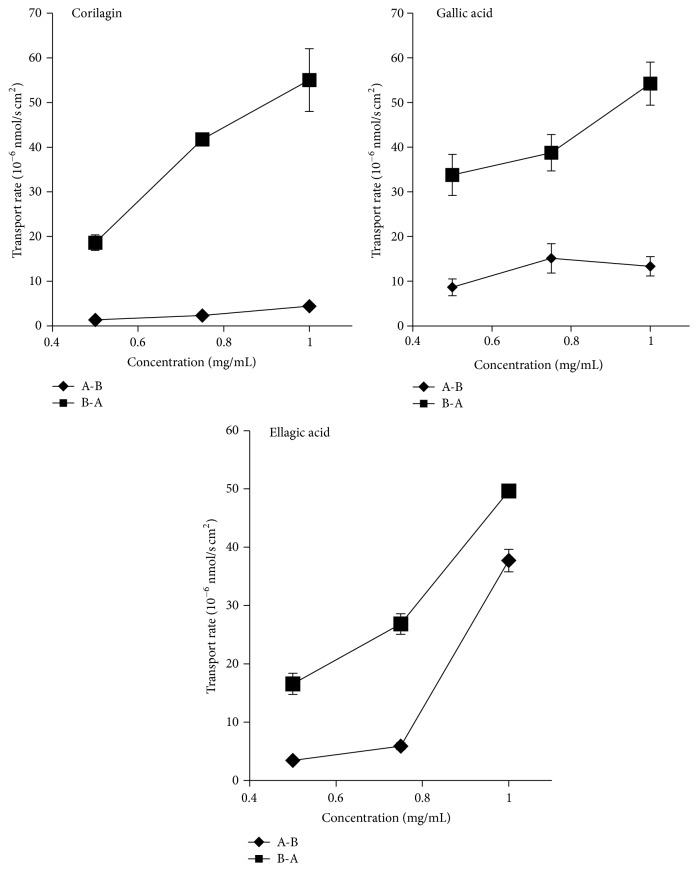
The transport rates of the transport of three compounds in PTF across Caco-2 cell monolayers with three concentrations. Caco-2 cells were incubated with PTF for 180 min. Results are expressed as the mean ± SEM for 3 determinations.

The Caco-2 cells were incubated at the concentration of 1.00 mg/mL for a period of time from 30 min to 180 min. Time courses of the absorption of PTF have been summarized in Figure 3. The transport rates of PTF of different concentration at 180 min are shown in Figure 4. As shown in Figures 3 and 4, the transport courses of all three compounds demonstrated that their transport percentages and transport rates were increased in a nonlinear manner with the time and concentration. These results suggested that the three compounds may be transported across the Caco-2 cell monolayers through protein mediated pathways.

### 3.5. Vectorial Characteristics of PTF Transport

The *P*_app_ values calculated for those constituents of PTF are presented in Table 2. The absorption transport *P*_app_ values of corilagin and EA were at the level of 10^−7^ cm/s, while that of GA was at the level of 10^−8^ cm/s. The secretion transport *P*_app_ values of GA and EA were at the level of 10^−7^ cm/s, while that of corilagin was at the level of 10^−6^ cm/s. We found statistically significant differences between absorption and secretion transport values. The PDR values of corilagin and GA were 12.52 and 4.17, respectively. The PDR levels suggested that the permeability of corilagin and GA in PTF was directional and could be affected by the efflux transport inhibitors.

### 3.6. Influence of Different Inhibitor on the Transport of PTF in the Caco-2 Cell Model

**Figure 5:**
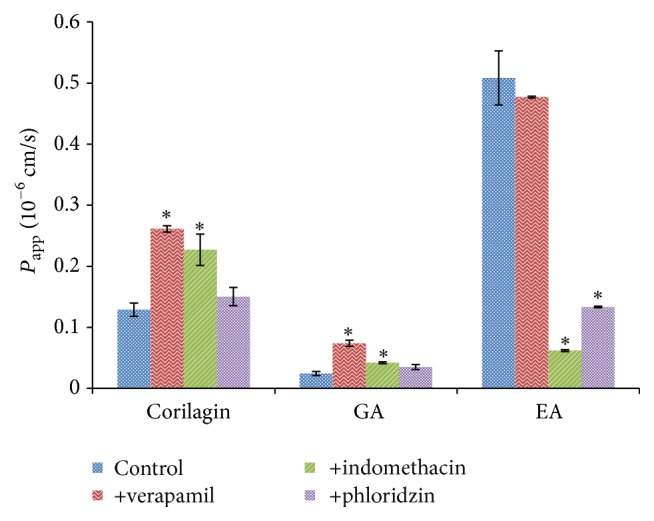
The effects of the transporters on the uptake of three compounds in PTF across Caco-2 cell monolayers. The concentrations of verapamil, indomethacin, and phloridzin were 100, 10, and 50 *μ*M, respectively. Caco-2 cells were incubated with PTF at the concentration of 1.00 mg/mL for 120 min. Results are expressed as the mean ± SEM for 3 determinations. ^*∗*^ *P* < 0.05 compared with the control group.

The effects of the transport inhibitors including verapamil, indomethacin, and phloridzin on the absorption of these compounds were showed in Figure 5. The transports of corilagin and GA were significantly increased when they were incubated with verapamil and indomethacin, but no considerable change was observed when they were incubated with phloridzin. The *P*_app_ value of EA was strongly decreased in the presence of indomethacin and phloridzin, while no substantial difference was detected in the presence of verapamil. These results indicated that the possible transport pathway of corilagin and GA from PTF may be a P-gp and MRPs mediated route in Caco-2 cells, and the transport of EA from PTF may be associated with OATP and SGLT1.

### 3.7. Epithelial Tight Junction Modulate Effect on PTF Transport

**Figure 6:**
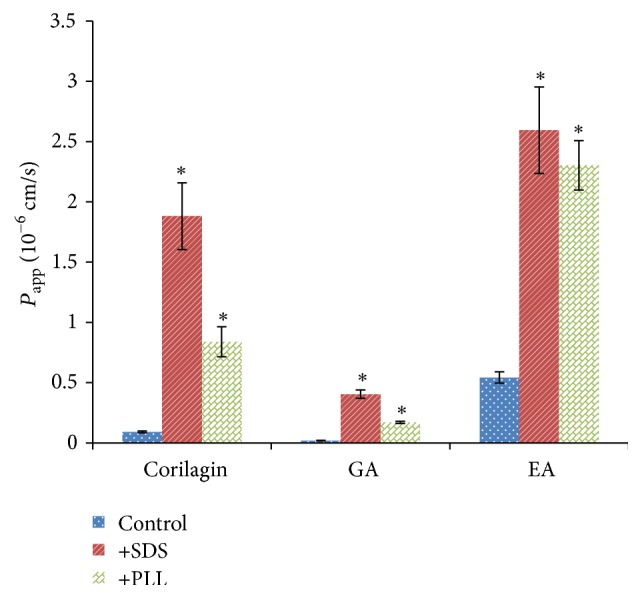
The effects of the tight junction modulators on the uptake of three compounds in PTF across Caco-2 cell monolayers. The concentrations of both SDS and PLL were 50 *μ*g/mL. Caco-2 cells were incubated with PTF at the concentration of 1.00 mg/mL for 120 min. Results are expressed as the mean ± SEM for 3 determinations. ^*∗*^ *P* < 0.05 compared with the control group.

As shown in Figure 6, the *P*_app_ values of the three compounds were enhanced 4.20- to 23.83-fold by SDS and PLL. These results suggested that the poor absorption of three compounds of PTF in Caco-2 cell monolayer may be due to the paracellular transport.

## 4. Discussion

Investigations of transport mechanism of herbal extracts across Caco-2 cell monolayer model have been widely concerned in recent years [34, 35]. In this study, the model was employed to investigate the intestinal absorption of the hydrolyzable tannin (corilagin) and its hydrolysates (GA and EA) from PTF. A preliminary analysis demonstrated that the concentrations of the three compounds after absorption were so low that they could not be detected by HPLC-UV method. LC-MS method was selected for its high sensitivity and selectivity, which was used in the analysis of transport of herbal extracts across Caco-2 cell monolayer model [36]. After incubation with Caco-2 monolayers for different periods of time from 30 min to 180 min, the three compounds from PTF appeared hardly absorbable as their *P*_app_ values were at the level or even lower than those of sodium fluorescein [32, 33]. These results were supported by our* in vivo* experiment and the prior reports: the absorption of EA was enormously poor after oral administration, and the *P*_app_ value of GA across Caco-2 cell monolayer was about 0.20 × 10^−6^ cm/s [37, 38].

The mechanism of the poor absorption of PTF was further investigated. According to Liu's report, the nonlinear time course of orientin and vitexin may be explained by the saturation of transporters [22]. The similar time course characters of corilagin and GA showed an uptrend of transport with incubation time increase, which demonstrated the contribution of the efflux transporters (Figure 3). In addition, the time course of EA reached a plateau with incubation time at 2 h, which indicated the participation of absorption transporters (Figure 3). When PDR was more than 1.5, the transport was considered to be directional [39]. The PDR levels of GA and corilagin were 4.17 and 12.52, respectively, which suggested the participation of efflux transport protein.

The P-gp, MRPs, and BCRP are efflux transporters; OATP, SGLT1, and MCT are uptake proteins. In this study, the transport of corilagin and GA involved P-gp and MRPs mediated active efflux in addition to passive diffusion, which was evidenced by their *P*_app_ values (less than 10^−6^ cm/s), PDR levels (more than 1.5) (Table 2), the nonlinear transport with time and concentration (Figures 3-4), and the significantly increased transport in the presence of verapamil and indomethacin. The transport of EA may be a little different, the PDR level was less than 1.5, and the absorption was obviously decreased in the presence of indomethacin and phloridzin, which demonstrated that EA was absorbed mainly through passive diffusion in combination with OATP and SGLT1 mediated active uptake. The transport of polyphenols was proved by previous reports to be mediated by both efflux and uptake proteins. The transport of resveratrol, apigenin, and puerarin was mediated by P-gp and MRPs [21, 35]; the uptake of chrysophanol and emodin was with the participation of SGLT1 [21]; the absorption of forsythoside A was participated by OATP [26]. The inhibitors of P-gp and MRPs but not SGLT1 or OATP may benefit the absorption of PTF* in vivo*.

The transepithelial transport of GA was reported earlier [38], and its *P*_app_ was about 0.20 × 10^−6^ cm/s, which was much higher than that of GA in PTF (0.024 × 10^−6^ cm/s). The absorption between pure compounds and that contented in herbal extracts appeared different, which was well proved in previous findings. As reported in the literature, the presence of other compounds in* Aconitum carmichaeli *[40] and* Schisandra chinensis *[41] extracts assisted the transport of aconitine and gomisin A, respectively. Besides, the components of apple peel extracts hindered the absorption of quercetin across the Caco-2 cells [34]. The interactions of other components in PTF on the absorption of GA should be considered in the next step.

Medium chain fatty acids (MCFAs) and polycations can enhance the absorption by affecting the tight junction permeability of Caco-2 cells [29, 30, 42]. SDS and PLL are typical MCFA and polycation, respectively. Both SDS and PLL were shown to affect intestinal epithelial monolayers differently in different time and dose. In our study, we tested the incubation time and dose and found that, before incubation with SDS (50 *μ*g/mL) and PLL (50 *μ*g/mL) for 30 min, the TEER values of monolayers were lower than 500 Ω/cm^2^ (250–350 Ω/cm^2^); after removing the SDS and PLL the TEER values could rise to 500–600 Ω/cm^2^ in two hours. Both SDS and PLL greatly improved the transport of corilagin, GA, and EA. Other tight junction modulators such as EDTA and sodium caprate could enhance the transport of 3,6′-disinapoylsucrose [28]. These results further suggested that the paracellular transport appeared to be main limiting factor for the uptake of three compounds in Caco-2 cell monolayers.

## 5. Conclusion 

This study indicated that the transports of the corilagin and its hydrolysates GA and EA from PTF were via passive diffusion combined with protein mediated transport. Specifically, the absorption of corilagin and GA was P-gp and MRPs mediated efflux, and the active uptake of EA was associated with the OATP and SGLT1 protein. The improvement of the transport of these compounds in PTF caused by transport protein inhibitors or epithelial tight junction modulators may be helpful for the increasing of oral absorption and improving of antitumor effect.

## Figures and Tables

**Table 1:** The intraday and interday precision and recovery of corilagin, GA, and EA in PTF.

Chemicals	Standard samples (ng/mL)	Intraday		Interday		Mean recovery (%)
		Mean (ng/mL)	RSD (%)	Mean (ng/mL)	RSD (%)	
Corilagin	32.65	39.48	3.44	38.24	4.48	117.12
	163.25	156.08	2.65	153.83	4.42	94.23
	326.50	308.63	3.22	301.89	4.11	92.46

Gallic acid	10.08	10.44	2.55	10.06	4.77	99.80
	50.40	47.25	3.26	47.05	3.37	93.35
	252.00	234.92	2.06	237.83	2.99	94.38

Ellagic acid	98.88	117.00	2.47	117.44	2.43	118.77
	494.40	519.71	1.97	503.26	4.01	101.79
	988.80	952.80	2.95	930.62	4.11	94.16

Recovery (%) = 100 × (amount found − original amount)/original amount.

**Table 2:** The bidirectional transport of corilagin, GA, and EA in PTF through Caco-2 monolayers.

Chemicals	*P*_app AP→BL_/10^−6^ cm/s	*P*_app BL→AP_/10^−6^ cm/s	PDR
Corilagin	0.174 ± 0.019	2.178 ± 0.228	12.52
Gallic acid	0.024 ± 0.004	0.100 ± 0.009	4.17
Ellagic acid	0.347 ± 0.018	0.456 ± 0.010	1.31

The *P*_app_ values were determined for a period of 180 min at the concentration of 1.00 mg/mL; results were expressed as the mean ± SEM (*n* = 3).
